# Local and systemic modulation of the PD-L1 pathway is a novel mechanism of Enzalutamide resistance in castration-resistant prostate cancer

**DOI:** 10.1186/2051-1426-2-S3-P94

**Published:** 2014-11-06

**Authors:** Jennifer Bishop, Alexander Sio, Arkhjamil Angeles, Morgan Roberts, Arun Azad, Kim Chi, Amina Zoubeidi

**Affiliations:** 1 Vancouver Prostate Centre, Vancouver, BC, Canada; 2 Department of Microbiology and Immunology, University of British Columbia, Vancouver, BC, Canada; 3 British Columbia Cancer Agency, Vancouver, BC, Canada

## 

The treatment effects of the anti-androgen Enzalutamide (ENZ) in patients with castration resistant prostate cancer (CRPC) are short lived. Immunotherapy may improve patient survival, however how efficacious these treatments are for CRPC, particularly those that inhibit T cell checkpoint molecules, remains questionable. Indeed, the lack of PD-L1 expression on CRPC tumors has made rationalizing the use of PD-1 blockade difficult for CRPC patients, however whether patients with ENZ resistant CRPC may be a more relevant cohort to examine the efficacy of anti-PD1 therapies remains unknown. In this study, we show that compared to CRPC, ENZ resistant CRPC expresses more PD-L1 in vitro and expresses high levels of both PD-L1 and 2 in vivo, suggesting that up-regulation of immune checkpoint molecules may be one unique mechanism of ENZ resistance that is not observed in CRPC. Our results also suggest that that ENZ resistant CRPC may not only be able to suppress immune responses via intrinsic PD-L1 expression, but also through the induction of PDL-1 and 2 on innate immune subsets in the circulation. This hypothesis was supported by our data showing an increased frequency of PD-L1/2 expressing dendritic cells (DC) and myeloid derived suppressor (MDSC) cells in the blood of ENZ resistant tumor bearing mice compared to those with CRPC. We also found that in vivo compared to CRPC, ENZ resistant CRPC was able to prevent DC infiltration into tumors and suppress DC activation, as marked by the reduced frequency of CD80/86 and PD-L1/2 expressing DC in the tumors. Moreover, we show for the first time that CRPC patients progressing on ENZ treatment have high frequencies of PD-L1^+ ^DCs and PD-1^+ ^CD8 T cells in their blood. Taken together, our work suggests that ENZ resistant CRPC in both mouse models and patients is associated with strong expression of the targets for anti-PD1 therapy. These data provide impetus for future studies that examine the relative contribution of tumor vs. immune cell PD-L1 in the progression of CRPC to anti-androgen resistance and the utility of monitoring circulating cell PD-L1 pathway activity in CRPC patients to predict responsiveness to checkpoint blockade immunotherapy.

